# Serum Occludin as a Biomarker to Predict the Severity of Acute Ischemic Stroke, Hemorrhagic Transformation, and Patient Prognosis

**DOI:** 10.14336/AD.2020.0119

**Published:** 2020-12-01

**Authors:** Weili Li, Zhifeng Qi, Huining Kang, Xuzhen Qin, Haiqing Song, Xueqin Sui, Yi Ren, Xunming Ji, Qingfeng Ma, Ke Jian Liu

**Affiliations:** ^1^Cerebrovascular Diseases Research Institute, Xuanwu Hospital of Capital Medical University, Beijing, China.; ^2^Department of Internal Medicine, University of New Mexico, Albuquerque, NM 87131, USA.; ^3^Chinese Academy of Medical Sciences & Peking Union Medical College Hospital, Beijing, China.; ^4^Department of Neurology, Xuanwu Hospital of Capital Medical University, Beijing, China.; ^5^Department of General Medicine, Affiliated Hospital of Weifang Medical College, Shandong province, China.; ^6^Department of Pharmaceutical Sciences, College of Pharmacy, University of New Mexico Health Sciences Center, Albuquerque, NM 87131, USA.

**Keywords:** occludin, blood-brain barrier, acute ischemic stroke, biomarker, hemorrhagic transformation, prognosis

## Abstract

Blood-brain barrier (BBB) damage plays an important role in overall brain injury following acute ischemic stroke (AIS). We investigated the potential utility of serum occludin, a BBB damage biomarker, in predicting the severity of AIS, hemorrhagic transformation (HT) and patient prognosis. A total of 243 patients, suspected of suffering an AIS and admitted to the emergency room at Xuanwu Hospital between November 2018 to March 2019, were enrolled in this study. Serum occludin levels were measured by enzyme linked immunosorbent assay and clinical data were collected from each patient. Receiver operating characteristic curves (ROC) were used to analyze the relationship between serum occludin and AIS. Multiple logistic regression analysis was used to analyze the relationship between serum occludin and stroke prognosis. Serum occludin levels were significantly elevated in acute stroke cases compared with those with stroke-like symptoms (P<0.001). In the moderate and severe cerebral infarction (CI) groups, serum occludin levels were significantly higher than those in the mild CI group (P<0.001). Patients with HT had higher occludin levels than non-HT patients (P<0.05). In addition, serum occludin level of patients with poor prognosis was significantly higher than that of the patients with good prognosis for non-reperfusion therapy. The ROC curve showed that serum occludin could reasonably predict HT and poor prognosis. Moreover, serum occludin were independently associated with 90-day poor prognosis. These findings suggest that the serum occludin levels could be used to identify early acute stroke cases and may predict the severity of AIS and HT as well as the prognosis at 90 days.

Stroke is a leading cause of death in the world, causing a substantial economic and social burden [1]. The long-term prognosis of acute ischemic stroke (AIS) and the resulting hemorrhagic transformation (HT) after thrombolysis and/or thrombectomy are major concerns. An acute stroke is usually evaluated and diagnosed by clinical symptoms and imaging evaluation. It may indicate a high risk of bleeding and a poor prognosis, if the patient has severe clinical symptoms and the imaging findings indicate a wide range of lesions [2]. However, the judgment of clinical symptoms is subjective, and it takes a long time to complete the magnetic resonance imaging (MRI) scan, which is not recommended in AIS patients. Therefore, an objective and accurate methodology is urgently needed to judge the severity of AIS, predict the risk of HT and determine the long-term prognosis before the clinical treatment initiation [3].

Blood biomarkers represent an objective measurement of molecular characteristics and have been proposed as a tool to help in stroke diagnosis. Studies of serum markers have been reported to reflect the involvement of various mechanisms in the occurrence of AIS, such as brain natriuretic peptide (BNP) suggesting a cardiogenic mechanism, matrix metalloproteinase-9 (MMP-9) showing inflammatory mechanisms, cellular fibronectin (cFn) as a marker of HT, S100 calcium binding protein B (S-100B) and neuron specific enolase (NSE) reflecting brain injury [4,5]. However, these markers are not specific for the diagnosis of AIS, because these markers can appear in a variety of diseases, such as tumor, encephalitis etc. Therefore, if a more specific serum marker that occurs only during acute stroke can be found, it will make a significant improvement in AIS assessment.

One of the pathophysiological features of ischemic stroke is disruption of the BBB, triggering and increasing the risk of cerebral ischemia, nerve damage and even cerebral edema and hemorrhage [6,7]. Occludin proteins are important components of tight junctions in the BBB and are implicated in the maintenance of their integrity [8]. From our animal and clinic studies [9,10], serum occludin has been shown to be a biomarker of BBB damage. Following the onset of cerebral ischemia, occludin protein is cleaved from the cerebral microvessels, some of which are released into the blood circulation, leading to increased serum occludin levels. Protecting the BBB resulted in reduced serum occludin level and decreased neurological deficits. However, it is still unclear what the specific relationship between serum occludin, HT and long-term prognosis is, and whether there is any difference in serum occludin level between stroke and stroke mimic patients. This study investigated whether this biomarker has any predictive value for the severity of a stroke, the resulting HT and the long-term prognosis.

**Figure 1.**
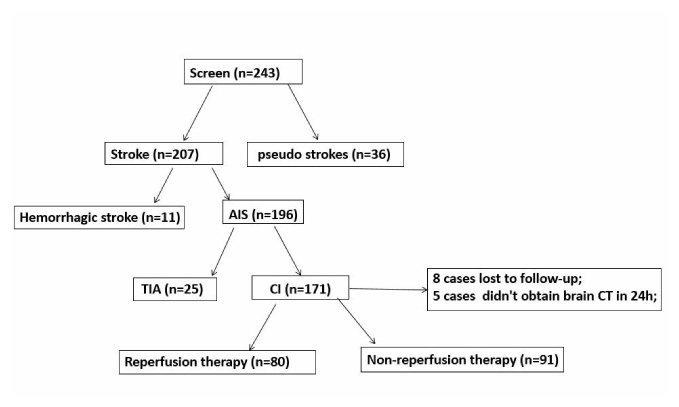
Patient screening flow chart. We screened 243 patients, including 207 stroke and 36 pseudo strokes patients. Stroke patients included 196 patients with AIS and 11 patients with cerebral hemorrhage. Among AIS patients, 171 were cerebral infarction (CI) patients and 25 TIA. For CI patients, 80 patients received reperfusion therapy, 91 patients received non-reperfusion therapy, 8 patients were lost in follow-up, and 5 patients did not complete 24-hour brain CT scan.

## MATERIALS AND METHODS

### Study Design and Participants

This study was approved by the ethics committee of Xuanwu Hospital, Capital Medical University. For this study, 243 patients with suspected acute strokes were diagnosed in the Department of Emergency Neurology at Xuanwu Hospital of Capital Medical University from November 2018 to March 2019. These patients were all aged 18 years and over, stroke onset was within 72 hours and all patients completed Computed Tomography (CT) within 90 minutes of admission. Informed consent was obtained for data analysis.

Patients were grouped according to clinical diagnosis and imaging results and divided into acute and pseudo stroke subgroups (Fig. 1). Cases of acute stroke were further classified as acute ischemic and acute hemorrhagic strokes. Based on the patient's clinical data, patients with cerebral infarction were stratified according to age, gender, presence of hypertension, diabetes, onset time, severity of disease, prognosis and HT.

### Stroke Diagnostic Criteria

The diagnostic criteria for AIS and hemorrhagic stroke: AIS mainly includes transient ischemic attack (TIA) and Cerebral infarction (CI). TIA is a brief episode of neurological dysfunction caused by a focal disturbance of brain or retinal ischemia, with clinical symptoms typically lasting less than 1 hour, and without evidence of infarction [11,12]. Hemorrhagic strokes include cerebral hemorrhage and subarachnoid hemorrhage. Pseudo strokes have the clinical manifestations of stroke episodes, but are ultimately confirmed as clinical syndromes of non-stroke causes such as epilepsy, syncope, periodic paralysis, etc.

**Table 1:** Baseline Characteristics of admitted patients.

Variable	Stroke group (n=207)	pseudo stroke (n=36)
Age — yr	65.6±13.2	58.2±14.2
Male sex — no. (%)	143 (69.1)	21 (58.3)
Atrial fibrillation — no. (%)	40 (19.3)	2 (5.6)
Coronary artery disease— no. (%)	32 (15.5)	4 (11.1)
Diabetes mellitus — no. (%)	60 (29.0)	10 (27.8)
Hypertension — no. (%)	125 (60)	22 (61.1)
Previous stroke — no. (%)	48 (23.2)	12 (33.3)
Median NIHSS score (IQR)	4 (3-5)	4 (2-7)
SBP (mmHg)	154.2±26.1	142.2±15.5
Median time from onset to puncture— hr (IQR)	4 (2.1-7.5)	3.8 (2.4-6.0)
LDL-C (mmol/L)	2.8±1.0	2.6±0.8
GLU (mol/L)	8.0±3.9	7.6±3.1
FIB (g/L)	3.6±1.1	3.6±1.0
Data were presented as means±SD or medians (IQR).		

### Clinical Data Collection

(1)General baseline information including demographic data, risk factors of cerebrovascular diseases, laboratory data etc (see Table 1); (ii) Disease assessment data including baseline National Institutes Health Stroke Scale (NIHSS) score, onset to treatment time, treatment method (non-reperfusion therapy or reperfusion therapy); (iii) Clinical prognostic assessment including mRS score at 90 days (see Prognostic evaluation) [13].

### Prognostic evaluation

Clinical prognostic assessment includes modified Rankin Scale (mRS) score at 90 days. The mRS scoring was applied according to the established guideline: 0, no symptoms at all; 1, no significant disability despite symptoms; 2, slight disability; 3, moderate disability; 4, moderately severe disability; 5, severe disability; 6, dead. In this study, a mRS score of 0~2 is consider good prognosis and 3~6 points as poor prognosis.

### Stroke Severity Evaluation

NIHSS score is normally assessed on emergency patients by two neurologists in the Emergency Access Department. If the two doctors disagreed, a senior doctor was required to confirm the verification to ensure the accuracy of the assessment. Stroke severity was assessed on the base of NIHSS score on hospital admission and was classified as mild (0-6), moderate (7-15) and severe (≥16) [14,15].

### Evaluation of Hemorrhage Transformation (HT)

Intracranial HT was evaluated at 22-36 hours after treatment to assess whether bleeding was found in the first cranial imaging examination, using CT or MRI after CI, either occurring naturally or if related to treatment [16]. It is based on the presence or absence of neurological deficits. These patients were further divided into symptomatic intracranial hemorrhage (sICH) and asymptomatic intracranial hemorrhage. According to the ECASS-III diagnostic criteria, intracranial hemorrhage (ICH) was confirmed by intracranial CT or MRI within 22-36 hours with an increase in NIHSS score≥4 [17].

### Treatment of AIS

Patients with AIS were divided into non-reperfusion therapy and reperfusion therapy according to indications for treatment and selection of patients. Non-reperfusion therapy involved general drugs such as anti-platelet, statin and Chinese medicine for promoting blood circulation and removing blood stasis and symptomatic treatment. Reperfusion therapy involved intravenous thrombolysis (IVT) and/or endovascular treatment (ET). Treatment choice was usually based on the patient's preferences and informed consent.

### Collection and Preservation of Blood Samples

After the patient arrived at the emergency room, blood (4 mL) was immediately collected by the emergency nurse (within 30 minutes) and then stood at room temperature for two hours. The sample was then centrifuged at 3,000 rpm for 10 min. The supernatant (serum) was collected and stored at -80°C for enzyme linked immunosorbent assay (ELISA) assay.

### Determination of Serum Occludin

The level of serum occludin was measured using a commercial Occludin ELISA Assay Kit (USCN, China), according to the manufacture’s instruction [16]. The standard curve and the regression equation were obtained according to the optical density value of standards. All tests were performed at the same time after the specimen was collected.

### Statistical Analyses

For continuous data, either means ± standard deviation or medians (interquartile range, IQR) were used to summarize data. A two-sided student’s *t*-test or a Mann-Whitney U test was performed to detect differences between groups. Differences among multiple groups were analyzed using one-way Analysis of Variance (ANOVA).

The effectiveness of baseline serum occludin for identifying acute stroke, severity of AIS and HT after CI, was evaluated using receiver operating characteristic (ROC) curve and the area under the curve (AUC). ROC curve was also applied to determine the optimal cut off point of serum occludin that distinguished between good and poor prognosis. According to the ROC curve, combined with the sensitivity and specificity results of each cut point, select the cut point on the curve as close as possible to the upper left Youden index as the optimal threshold value. The Youden index is the sum of sensitivity and specificity minus 1. In addition, Spearman's rank correlation coefficient was used to analyze the relationship between occludin and 90-day mRS score. The correlation between serum occludin and 90-day mRS was further investigated according to different groups of disease severity. All data was analyzed using an SPSS 23.0 (IBM Corporation, Armonk, NY, USA) with the significance level set at α=0.05 (two sided tests).

## RESULTS

The diagnosed 243 patients include 207 cases of stroke (196 cases of ischemic stroke; 11 cases of hemorrhagic stroke); 36 patients with pseudo strokes which mainly includes hypoglycemia, traumatic brain injury, encephalitis, somatization disorders, etc. Among the 196 cases of ischemic stroke are 171 cases of cerebral infarction (CI) and 25 cases of transient ischemic attack (TIA) (see Figure 1 for details). Baseline features of strokes and pseudo strokes are shown in Table 1.

### AIS and the Level of Serum Occludin

**Figure 2.**
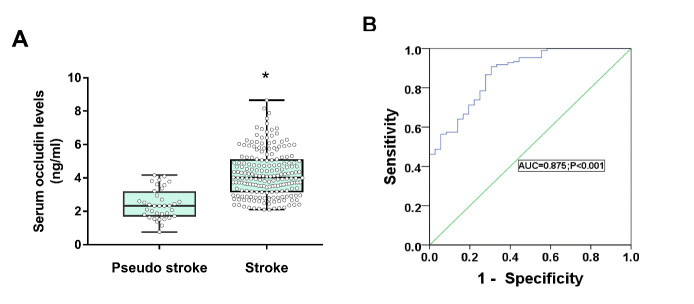
Differences of serum occludin level in stroke and pseudo stroke. (A) Baseline serum occludin levels were elevated in the stroke group compared with the pseudo stroke group; Data are expressed as the mean±SD, ^*^P<0.05. (B) ROC curve for serum occludin on predicting stroke.

To explore the relationship between serum occludin and acute stroke, patients were divided into acute and pseudo strokes groups and the mean differences in serum occludin levels were compared. The results showed that baseline serum occludin levels were significantly elevated in the acute stroke group compared with the pseudo strokes group (4.24±1.37 ng/mL vs 2.36±0.96 ng/mL, P<0.001; Fig. 2A, Table S1). The ROC curve analysis showed that baseline serum occludin was significantly predictive of stroke occurrence, with an AUC value of 0.875 (95% confidence interval: 0.815-0.934, p<0.001), with a threshold of 2.61 ng/mL (Fig. 2B). This result suggests that unlike pseudo strokes, acute strokes caused elevated level of serum occludin, as a results of BBB damage.

### Differences in serum occludin levels in stroke subtypes

**Figure 3.**
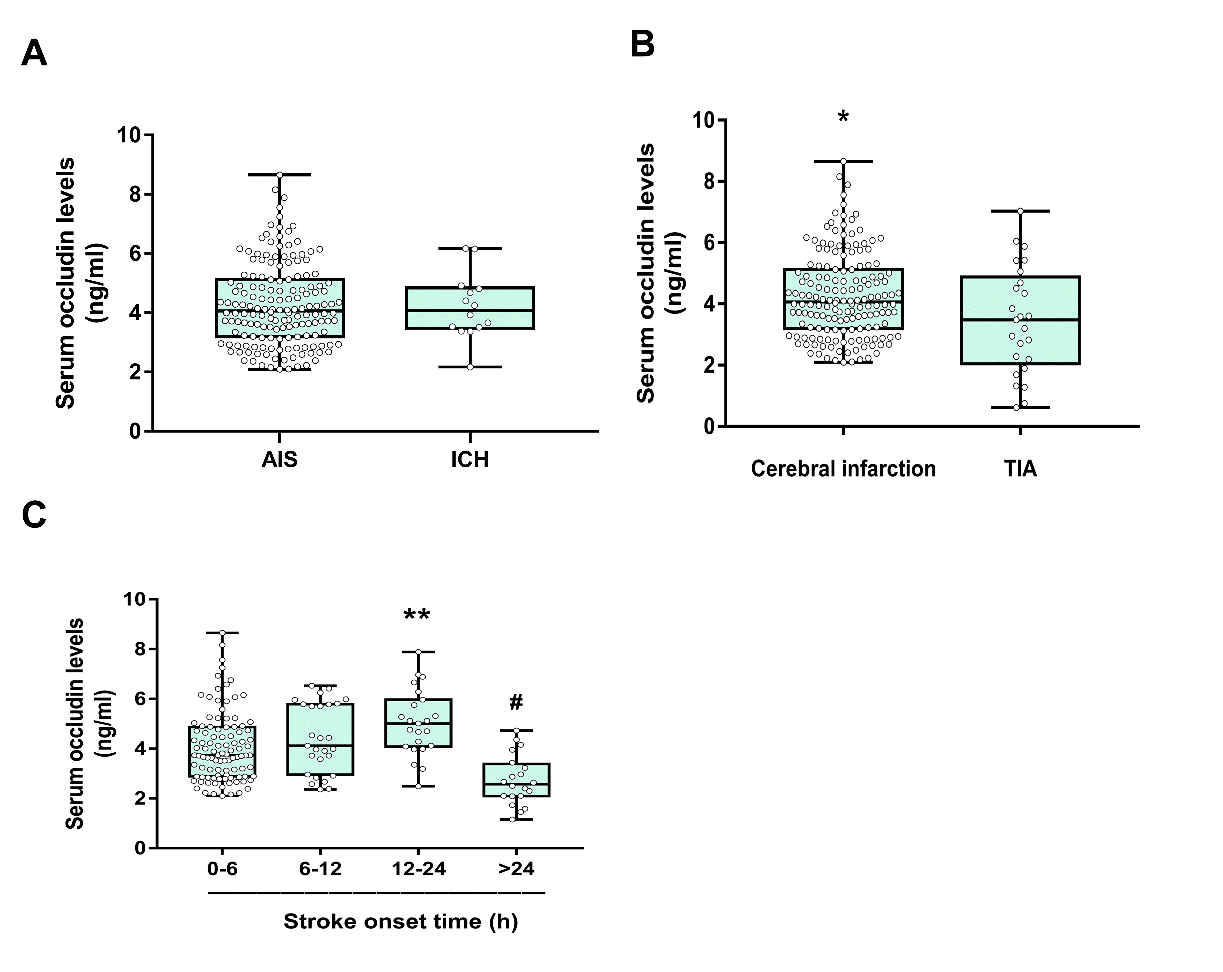
Comparison of serum occludin between different stroke subgroups. (A) Mean levels of serum occludin between AIS and ICH groups were not significantly different; (B) Comparison of the mean baseline serum occludin concentrations between the CI and TIA groups (*P<0.05); (C) Serum occludin concentration gradually increased with the prolongation of onset time, peaking at 12 to 24 hours; data are expressed as the mean±SD, ^**^P<0.001 (the occludin levels in the 24 hour group was significantly lower than other groups (P<0.001); ^#^P<0.05 (the occludin levels in the 12-24 hour group were higher than the 0-6 hour group).

We further investigated the difference of serum occludin levels between AIS and hemorrhagic strokes. The data showed that the level of serum occludin was not significantly different in the two groups (Fig. 3A, Table S1), implying that the level of serum occludin did not seem to distinguish the ischemic stroke patients from hemorrhagic stroke patients.

In order to further clarify whether the serum occludin levels were the same in the CI and TIA, ischemic stroke patients were divided into two groups. The data demonstrated that the level of serum occludin in CI group was significantly higher than TIA group (4.22±1.41 vs 3.46±1.75 ng/mL, P<0.05, Fig. 3B and Table S1). This result suggested that the level of serum occludin may distinguish the CI patients from TIA patients.

### Diabetic and Elderly Patients and the Effect on Serum Occludin

To analyze the effects of risk factors for CI on BBB injury, a subgroup analysis of CI by age, gender, hypertension, and diabetes was performed. The results showed that serum occludin levels were slightly different in four age groups (P=0.022). The serum occludin concentration was a little higher in the over 80 years group (4.90±1.51 ng/mL) and higher in the diabetic group compared with the nondiabetic group (4.59±1.28 ng/mL vs 4.03±1.24 ng/mL, P=0.013). Hypertension and gender had no significant effect on baseline serum occludin levels (see Table 2). These findings suggest that diabetes and advanced age may be weak risk factors for BBB injury, while hypertension and gender may not be significant.

**Table 2:** Subgroup analysis of Serum occludin levels in CI patients.

Subgroup	n	Serum occludin (ng/ml)	P values
Age (yr)			
All	171		0.029^a^
0-45	13	3.49±1.00	
46-65	74	4.24±1.29	
66-79	55	4.14±1.43	
>80	29	4.90±1.51	
Sex			
All	171		0.639
Male	124	4.23±1.34	
Female	47	4.35±1.53	
Diabetes			
All	171		0.013
Non-Diabetes	118	4.03±1.24	
Diabetes	53	4.59±1.28	
Hypertension			
All	171		0.66
Non-hypertension	61	4.21±1.53	
Hypertension	110	4.31±1.31	

a There were statistically significant differences between the four groups (P=0.029). Pairwise comparison showed that only elderly patients over 80 years old had statistical significance compared with other age groups (P <0.05). One-way Analysis of Variance (ANOVA) was applied.

### CI Patients and Serum Occludin Trends

In order to clarify the relationship between serum occludin and the ischemic duration, patients were classified into four groups according to time interval from the onset of stroke symptoms to the arrival of the hospital (0-6 h, 6-12 h, 12-24 h, >24 h). Serum occludin concentration in 12-24h group was higher than 0-6 h group (5.08±1.45 ng/mL vs 4.13±1.40 ng/mL). The level of serum occludin reached to a peak in 12-24 h group and then decreased in >24 h group (see Fig. 3C and Table S2). This indicated that the serum occludin level gradually increased with the extension of ischemic time and reached a peak in 12- 24 hours.

### Serum Occludin Levels in CI

**Figure 4.**
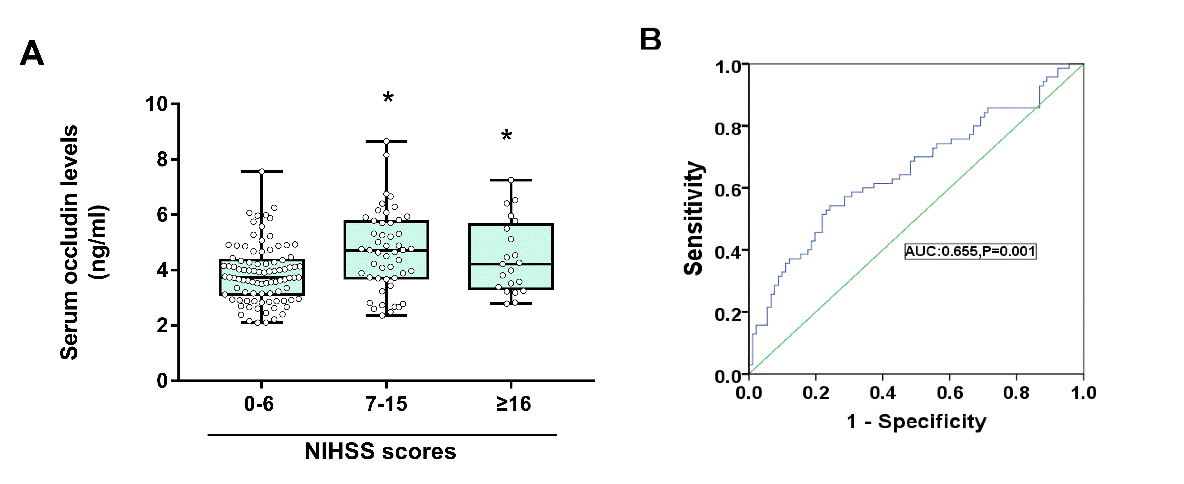
The relationship between serum occludin levels and CI severity. (A) Serum occludin concentrations in patients with moderate and severe CI were significantly increased compared with those with mild CI (P<0.001); data were expressed as the mean±SD, ^*^P<0.05. (B) ROC curve for serum occludin on predicting severity of CI.

To investigate the differences in serum occludin levels between different degrees of stroke induced neurological impairment, the patients were divided into three groups based on NIHSS scores: mild (0-6), moderate (7-15) and severe cases (≥16). The concentrations of occludin were significantly lower in the mild group than moderate and severe groups (P<0.001; Table S2, Fig. 4A). After combining the moderate and severe groups, the ROC curve analysis showed that the high baseline serum occludin was indicative of moderate to severe CI (AUC = 0.655, 95% confidence interval: 0.568-0.742, P<0.001, Fig. 4B) and a threshold value of 4.36 ng/mL. This showed that BBB destruction was related to the severity of the disease.

### Serum Occludin and the Risk of HT

We also analyzed the serum occludin level in the cerebral infarction patient (n=171), which included 19 with intracranial HT and 147 without intracranial HT. Five additional patients did not obtain CT scan and were not included in the calculation. We considered the general baseline data of patients in the two groups, and found no significant difference in age, gender, previous risk factors (such as hypertension, diabetes, atrial fibrillation and history of stroke), average time from onset to door, and proportion of IVT (p>0.05); except for the NIHSS score (12 vs 5, P<0.001) between the two groups (See Table 3). In order to eliminate the effect of different treatment methods on patient prognosis and HT, we divided these patients into non-reperfusion therapy and reperfusion therapy groups.

**Table 3:** Baseline characteristics of HT and non-HT groups in CI patients.

Variable	HT (n=19)	Non-HT (n=147)	P values
Age — yr	68.68±12.49	66.15±12.77	0.416
Male sex — no. (%)	16 (84.2)	107 (72.8)	0.285
Atrial fibrillation — no. (%)	6 (31.6)	32 (21.8)	0.338
Diabetes mellitus — no. (%)	6 (31.6)	40 (27.2)	0.689
Hypertension — no. (%)	7 (36.8)	60 (40.8)	0.740
Median NIHSS score (IQR)	12 (6-15)	5 (2-11)	<0.001
Previous stroke — no. (%)	5 (26.3)	30 (20.4)	0.552
Median Onset to Door time— hr. (IQR)	5 (2.5-8)	4.1 (2.3-7)	0.352
IVT therapy	7 (36.8)	45 (30.6)	0.582
GLU (mmol/L)	9.05±3.25	8.48±4.09	0.673
FIB (g/L)	3.0±0.89	3.43±1.07	0.232

Data were presented as means±SD or medians(IQR).

#### A. Non-reperfusion therapy Group

**Figure 5.**
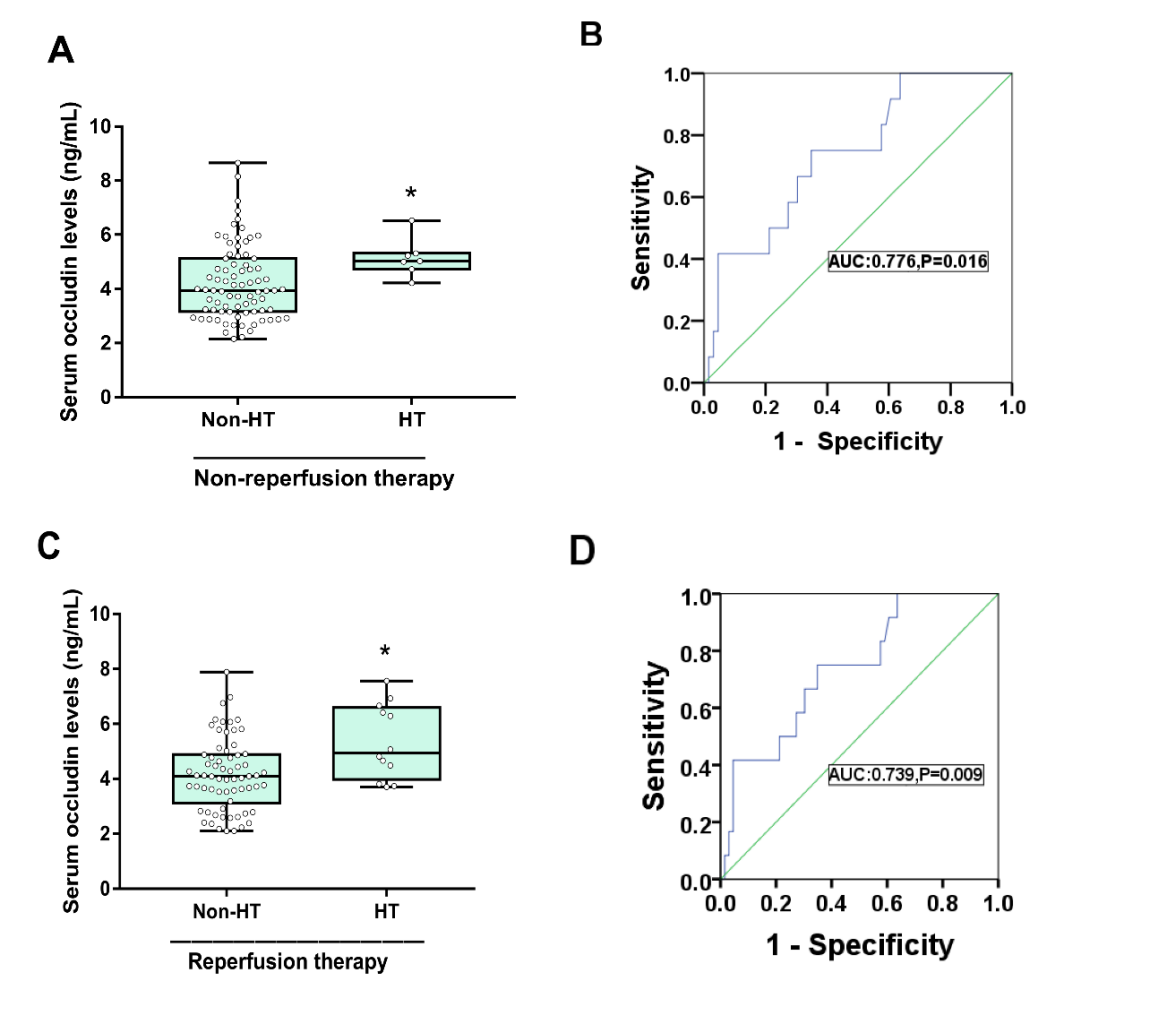
Differences of serum occludin in HT and non-HT in AIS patients. Patients with HT had higher serum occludin levels than patients without bleeding for non-reperfusion therapy (A) and for reperfusion therapy (C); Data is expressed as the mean±SD, ^*^P<0.05. ROC curve for serum occludin on predicting HT for patients of non-reperfusion therapy (B) and for patients of reperfusion therapy (D).

Intracranial HT occurred in seven of the 85 patients (8.2%) in the non-reperfusion therapy group, while none of the patients appeared symptomatic intracranial hemorrhage. Patients with HT had significantly higher baseline occludin levels (5.15±0.71 ng/mL vs 4.11±1.20 ng/mL, P=0.029; Table S2, Fig. 5A). The ROC curve showed that serum occludin could reasonably predict HT with an AUC of 0.776 (95% Confidence interval: 0.664-0.887, P=0.016). There was a threshold value of 4.18 ng/mL (Fig. 5B).

#### A. Reperfusion therapy

Among the 78 reperfusion therapy patients, 12 underwent HT (15.3%) and symptomatic intracranial hemorrhage occurred in two patients (2.6%). The serum occludin level in the HT group was significantly higher at 5.34±1.36 ng/mL compared with the non-HT group (4.16±1.31 ng/mL, P=0.005, Table S2, Fig. 5C). The ROC curve showed an AUC value of 0.739 (95% confidence interval: 0.595-0.884, P=0.009). The threshold value was 4.48 ng/mL (Fig. 5D).

The above results indicate that patients with increased serum occludin have a high risk of HT, regardless of non-reperfusion therapy or reperfusion therapy, suggesting that it may be a sensitive marker for prediction of intracranial hemorrhagic transformation.

### Serum Occludin Levels and Prognosis

Patients of non-reperfusion therapy (n=85) and reperfusion therapy (n=78) were further divided into a good prognosis (mRS: 0-2) and poor prognosis subgroup (mRS: 3-6) according to their 90 days prognosis. The difference of serum occludin level in these subgroups were observed. 8 patients were lost to follow up (Fig. 1).

#### A. Non-reperfusion therapy

**Figure 6.**
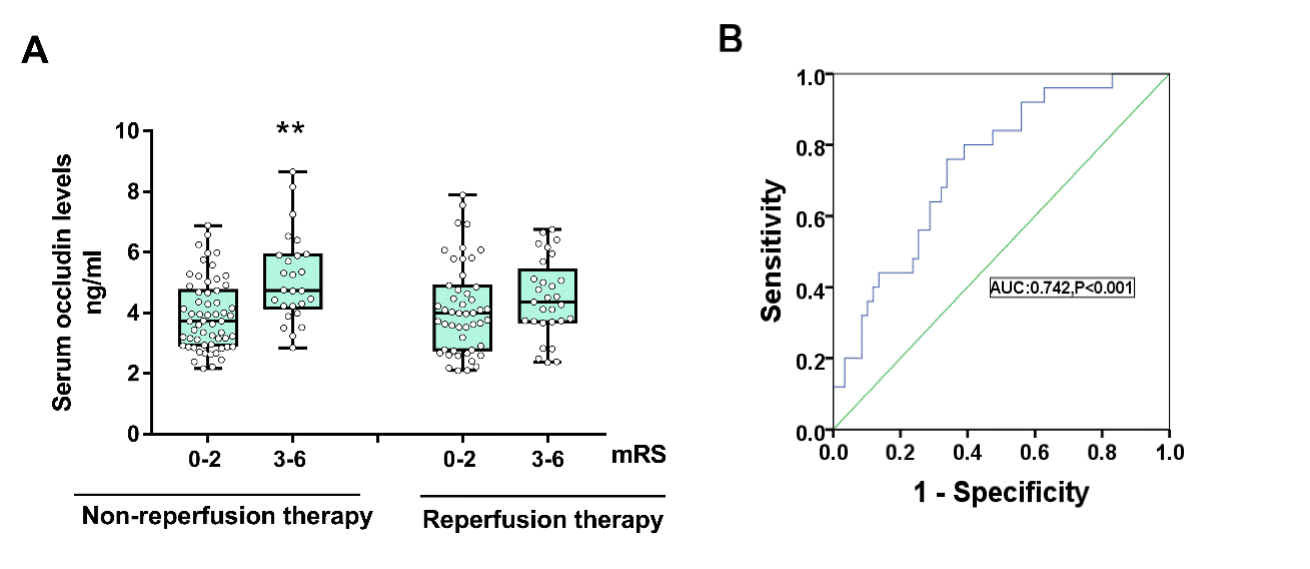
Comparison of serum occludin levels between good and poor prognosis groups. (A) Baseline serum occludin levels was higher in patients receiving non-reperfusion therapy with poor prognosis than that in patients of good prognosis (P<0.001); however, it was not statistically significant in reperfusion therapy (P=0.275). Data are expressed as the mean±SD, ^**^P<0.001. (B) ROC curve for serum occludin on predicting poor outcome for CI in non-reperfusion treated patients.

There were 59 patients with good prognosis and 26 patients with poor prognosis. The baseline characteristics of the two subgroups are shown in Table 4. Statistical analysis showed that there were no significant differences in age, sex, hypertension, diabetes, blood glucose, and blood lipids between the two subgroups (P>0.05). Compared with the good prognosis subgroup, the poor prognosis subgroup had a significantly higher serum occludin levels (5.11 ±1.48 ng/ml vs 3.93 ±1.16 ng/ml, P<0.001) (Fig. 6A). Additionally, the poor prognosis subgroup had a higher NIHSS score (12 vs 2, P<0.001), higher white blood cell count (mean 9.01 vs 7.28, P=0.002), higher percentage of neutrophils (74.51% vs 63.43%, P=0.001), higher rates of atrial fibrillation (26.9% vs 6.8%, P=0.028), and higher fibrinogen levels (mean 4.30 vs 3.61, P=0.007) (see Table 4).

**Table 4:** Analysis of prognostic risk factors for CI with non-reperfusion therapy.

Variable	mRS=0-2 (n=59)	mRS≥3 (n=26)	P values
Age — yr	66.46±13.34	68.32±14.76	0.570
Male sex — no. (%)	43 (72.9)	17 (65.4)	0.284
Atrial fibrillation — no. (%)	4 (6.8)	7 (26.9)	0.028
Diabetes mellitus — no. (%)	13 (22.0)	4 (15.4)	0.680
Hypertension — no. (%)	25 (42.4)	15 (57.7)	0.192
Median NIHSS score (IQR)	2 (1-4)	12 (10-14)	0.000
Previous stroke — no. (%)	10 (9.3)	7 (12.7)	0.493
Median Onset to Door time— hr. (IQR)	5 (2.5-12)	5.75 (4.5-9.75)	0.509
Serum occludin levels (ng/mL)	3.93±1.16	5.11±1.48	<0.001
LDL-C (mmol/L)	2.77±1.02	3.06±0.97	0.266
HDL-C (mmol/L)	1.27±0.37	1.23±0.35	0.683
TG (mmol/L)	1.79±1.10	1.77±0.86	0.938
GLU (mmol/L)	7.58±3.74	7.61±3.18	0.969
FIB (g/L)	3.61±0.80	4.30±1.27	0.007
WBC (10^9^/L)	7.28±2.18	9.01±1.96	0.002
NEUT (%)	63.43±11.42	74.51±13.98	0.001

Data were presented as means±SD or medians (IQR).

Results from univariate analysis revealed that serum occludin was associated with poor prognosis at 90 days (mRS≥3) (unadjusted odds ratio: 2.40, 95% confidence interval: 1.53-3.77; P<0.001). Further multivariate regression analysis was performed after adjusting for age, gender, atrial fibrillation, NIHSS scores, fibrinogen, and white blood cell counts. It was found that serum occludin levels were independently associated with 90-day poor prognosis (mRS≥3) (adjusted odds ratio: 2.46, 95% confidence interval: 1.17 -5.17; P=0.018) (see Table S3).

The ROC curve showed that baseline serum occludin had a predictive power for 90-day prognosis of an AUC value of 0.742 (95% Confidence interval: 0.632 to 0.852, P<0.001) with a threshold value of 4.18 ng/mL (Fig. 6B).

#### B. Reperfusion therapy

For the 78 patients with reperfusion therapy, there was no significant difference among the measured parameters between the good prognosis and the poor prognosis group (see Table S2 and Fig. 6A).

Finally, we combined with NIHSS score to explore the correlation between serum occludin and the 90-day prognosis of stroke. First, Spearman's rank correlation coefficient was used to analyze the correlation between occludin and 90-day mRS, which was found to have a very weak correlation between them (r=0.281, P<0.001). Then we proceed to the subgroup analysis, classified as mild (0-6), moderate (7-15) and severe (≥16) groups on the base of NIHSS score on hospital admission [12,13]. We found that they are significantly correlated (r=0.635, P<0.001) in severe group (NIHSS≥16). However, no correlation was found between the mild group (NIHSS=0-6) and the moderate group (NIHSS=7-15) (r=0.054, P=0.612; r=0.18, P=0.211). This indicates that the correlation between serum occludin and 90-day mRS score was stronger for patients with severe CI (NIHSS≥16).

The above results showed that elevated serum occludin levels were associated with prognosis at 90 days, and that serum occludin seems to be a good marker for predicting prognosis that needs further verification in the future.

## DISCUSSION

This is the first significant clinical study to explore the relationship between BBB injury, serum occludin, HT and long-term prognosis in AIS. Since AIS-induced BBB damage results in abnormally elevated serum occludin, early detection of serum occludin levels to identify BBB injury could provide an important basis for brain protection that cannot be identified by imaging evaluation in the early stages of BBB damage.

It is known that the degree of BBB disruption will influence the severity of cerebral edema, HT and neurological damage [18]. And these alterations could be potentially determined or predicted by measuring the serum occludin level. The results of this study show that serum occludin was elevated in patients with moderate to severe neurological deficits. The ROC curve shows that the baseline serum occludin had a weak ability to predict the degree of disease. The study provided a better reference value to assess the severity of the disease for those patients who cannot cooperate to complete a neurological test.

HT is one of the most common complications after IVT and ET in CI that can strongly affect functional recovery or early death [19]. Previous studies have shown that the underlying mechanism leading to extravasation is the destruction of the BBB [19] and IVT or ET can aggravate the destruction of the BBB and increase HT risk [20]. Early identification of patients with high intracranial HT risk may reduce the incidence of thrombolytic bleeding with an improved prognosis and the reduction of mortality. This study showed that patients with HT had significantly higher serum occludin levels than those without bleeding, with both non-reperfusion and reperfusion therapy. The ROC curve also confirmed that baseline serum occludin levels had a predictive value for HT. Although the sensitivity and specificity are not very high, which could be further improved by developing more sensitive and/or specific methods to detect serum occludin, this study establishes the proof of principle for utilizing occludin level for the prediction of HT.

The BBB is damaged as an early critical event following ischemic stroke that causes edema formation, initiation of the inflammatory cascade and ultimately the most serious outcomes of HT [21]. Theoretically, the greater the damage to the BBB, the more severe the disease and the worse the prognosis. The serum occludin level may be able to reflect the extent of BBB injury that affects the long-term prognosis for stroke patients [9]. Our previous study suggested that normobaric oxygen could protect the BBB, reducing the level of serum occludin after a stroke and improving the patient's 7-day NIHSS score [10]. The current study shows that patients in the non-reperfusion therapy group with a higher level of baseline serum occludin had a worse 90-day prognosis (mRS≥3). Furthermore, Spearman correlation analysis indicates the correlation between serum occludin and 90-day mRS score was stronger for patients with severe CI (NIHSS≥16). This fully demonstrates the predictive value of occludin as a marker for 90 days clinical outcome. Based on the NIHSS score and occludin level on admission, the assessment of stroke prognosis will be more accurate and reliable, and it needs to be confirmed by further prospective cohort studies.

However, there is no significant difference between the two groups for patients with reperfusion therapy (IVT or ET) in this study. We speculate that the BBB and brain tissue are protected in time because of recanalization. Therefore, the initial higher serum occludin levels may not be a major risk factor affecting the prognosis of patients with reperfusion therapy. More importantly, for patients with non-reperfusion therapy, if the blood vessels are not recanalized, cerebral ischemia and the continued hypoxia may result in serious BBB damage and irreversible change, which corresponds to significant increases of serum occludin. Therefore, the destruction of the BBB may be a key factor affecting the prognosis of AIS with non-reperfusion therapy. Finding from our results suggest that if the BBB can be given earlier protection, it may improve the patient's long-term prognosis. Obviously, further clinical trials are required to validate this notion.

Our previous studies have suggested that serum occludin levels can reflect the destruction of the BBB after stroke [9,10] and act as a specific marker of BBB injury [22]. This study confirmed that serum occludin level in the acute stroke group was significantly higher than that of the pseudo stroke group, which reflected the close relationship between BBB injury and acute stroke. Although the serum occludin level was reduced in the >24h group in our study, it did not necessarily mean that BBB’s damage was reduced after 24 hours. In fact, at early stage of AIS (<24 hours after stroke onset), serum occludin level reflects the severity of BBB damage, since degraded occludin is directly associated with BBB damage. After 24 hours, it will become more complicated due to multiple ongoing processes, including metabolism and excretion of occludin fragment, as well as repair of BBB damage, etc. As a result, level of occludin may not be directly related to the severity of BBB damage. Furthermore, in the > 24-hour group, the time span of onset of the patients was large and the onset time of most patients was about 7 days, only 4 cases occurred within 24-72 hours. Therefore, it did not fully reflect the changes in serum occludin levels after 24h. In the future, a prospective study may be needed to subdivide patients with onset more than 24 hours into 24-72h group, 72h-7days group, and >7days group to observe the changes of serum occludin levels in different time periods.

Our study also found differences in serum occludin levels between CI group and TIA group (P<0.05). Permanent cerebral ischemia is more pronounced than transient cerebral ischemia, which is consistent with previous findings [18,23]. It may be concluded that BBB injury is reversible like brain tissue damage when cerebral ischemia is transient. Interestingly, diabetes and advanced age can cause a slight increase in serum occludin levels. A variety of studies have shown that diabetes can weaken the BBB [24,25]. This study supports this finding by showing that serum occludin levels in the diabetes group were slightly higher than the non-diabetic group. In addition, this study shows that serum occludin concentrations in elderly stroke patients were higher than for other age groups, confirming that old age may be a risk factor for BBB injury. However age was not an independent factor, because advanced age was associated with factors such as hypertension, diabetes, and cerebral ischemia, which are known to disrupt the integrity of the BBB [26].

### Limitations of the Study

First, lack of strict inclusion and exclusion criteria meant that the study group could not be homogenized and there may be undetermined confounding factors influencing the false positive rate of results. Second, due to the small number of patients with onset of 24-72h hours, we did not subdivide them, but grouped them into the> 24h group, which is also a shortcoming of this study. Further prospective cohort studies may be needed to analyze the serum occludin levels at different times and between different stroke subgroups. Finally, we only observed intracranial HT at 22-36h after treatment. Because we included emergency patients and the data information was limited, we could not obtain the results of the patient's imaging examination at 1 week, which is also another potential deficiency of this study. A prospective cohort study will be conducted in the future to further confirm the results.

### Conclusions

The baseline serum occludin level can aid in the identification of early acute strokes and might act as a sensitive predictor of the extent of acute ischemic stroke, HT and 90 days prognosis. A larger prospective cohort study is needed in the future to validate these conclusions.

## Supplementary Materials

The Supplemenantry data can be found online at: www.aginganddisease.org/EN/10.14336/AD.2012.0119.
