# Supplementary material for: Serum Occludin as a Biomarker to Predict the Severity of Acute Ischemic Stroke, Hemorrhagic Transformation, and Patient Prognosis
Source: Aging Dis. 2020 Dec 1;11(6):1395–406. doi: 10.14336/AD.2020.0119 (PMC7673856; doi:10.14336/AD.2020.0119)
Supplement: Supplementary file 1 — The Supplemenantry data can be found online at: www.aginganddisease.org/EN/10.14336/AD.2012.0119. [file AD-11-6-1395-s.pdf]

SUPPLEMENTARY DATA

Serum Occludin as a Biomarker to Predict the Severity of Acute Ischemic Stroke, Hemorrhagic Transformation, and Patient Prognosis

Weili Li[1], Zhifeng Qi[1], Huining Kang[2], Xuzhen Qin[3], Haiqing Song[4], Xueqin Sui[5], Yi Ren[4], Xunming Ji[1], Qingfeng Ma[4*], Ke Jian Liu[6*]

© 2020. Li W et al. Published online at http://www.aginganddisease.org/EN/10.14336/AD.2020.0119

Supplementary Table 1. The comparison of serum occludin level in stroke patients.

Subgroup	n	Serum occludin (ng/ml)	P values
**Type of disease**	243		
Stroke	207	4.24±1.37	<0.001
Pseudo stroke	36	2.36±0.96	
**Type of stroke**	207		
Ischemic stroke	196	4.21±1.40	0.971
Hemorrhagic stroke	11	4.23±1.37	
**Type of ischemic stroke**	196		
Cerebral infarction	171	4.22±1.41	0.017
Transient ischemic attack	25	3.46±1.75	

Data were presented as means ± SD.

Supplementary Table 2. Subgroup analysis of Serum occludin levels in CI patients.

Subgroup	n	Serum occludin (ng/ml)	P values
Stroke onset time－h			
All	171		<0.001[a]
0-6	101	4.13±1.40	
6-12	27	4.41±1.38	
12-24	23	5.08±1.45	
>24	20	2.77±1.04	
NIHSS score on admission			
All	171		<0.001[b]
NIHSS(0-6)	96	3.88±1.05	
NIHSS(7-15)	54	4.80±1.38	
NIHSS(≧16)	21	4.56±1.31	
Hypertension	110	4.31±1.31	
Hemorrhagic transformation [#]			
Non-reperfusion therapy			
All	87		0.029
Hemorrhage	7	5.15±0.71	
Non-hemorrhage	80	4.11±1.20	
Reperfusion therapy			
All	79		0.005
Hemorrhage	12	5.34±1.36	
Non-hemorrhage	67	4.16±1.31	
Prognosis at 90 days*			
Non-reperfusion therapy			
All	85		<0.001
Good prognosis (mRS:0-2)	59	3.93±1.16	
Poor prognosis (mRS:3-6)	26	5.11±1.48	
Reperfusion therapy			
All	78		0.275
Good prognosis (mRS:0-2)	49	4.14±1.48	
Poor prognosis (mRS:3-6)	29	4.51±1.31	

a There were statistically significant differences among the four groups (P<0.001).Two-two comparison found that serum occludin levels in the group over 24 hours was significantly reduced compared with other groups (P<0.05); Serum occludin in 12-24 hours

© 2020. Li W et al. Published online at http://www.aginganddisease.org/EN/10.14336/AD.2020.0119

group was significantly increased compared with that in 0-6 hours group (P=0.01). One way Analysis of Variance (ANOVA) was applied.

b There were statistically significant differences among the three groups (p<0.001). Pairwise comparison showed that the moderate and severe groups had statistical significance compared with the mild group (P<0.05, respectively).One way Analysis of Variance (ANOVA) was applied.

[#] Five of the 171 patients were unable to identify intracranial hemorrhage due to discharge within 24h.
* Eight of the 171 patients were lost to follow-up.

Supplementary Table 3. Regression analysis between serum occludin and 90-day stroke prognosis.

Variable	Unadjusted OR (95%CI)	Adjusted OR (95%CI)	Unadjusted/adjusted P
Serum occludin levels	2.40 (1.53-3.77)	2.46 (1.17 -5.17)	<0.001/0.018
Median NIHSS score	1.47 (1.26-1.72)	1.37 (1.15-1.62)	<0.001/<0.001
FIB	2.04 (1.15-3.62)	1.97 (1.15-5.16)	0.015/0.073
White blood cell count	1.44 (1.12-1.85)	1.19 (0.80-1.75)	0.004/0.394

© 2020. Li W et al. Published online at http://www.aginganddisease.org/EN/10.14336/AD.2020.0119
